# Bactericidal and anti-inflammatory activity of defined hypochlorite solution

**DOI:** 10.1371/journal.pone.0352576

**Published:** 2026-07-24

**Authors:** Christopher David Chapman, Myles Hyla Edward Dakin, Richard Aspinall

**Affiliations:** 1 Centre for Intelligent Healthcare, Coventry University, United Kingdom; 2 Hypo-stream Limited, Cambridge, United Kingdom; Universiti Teknologi Malaysia, MALAYSIA

## Abstract

**Background:**

Hypochlorite is an antiseptic that has been used for over a century for industrial sterilisation and antisepsis. More recently, the topical application of hypochlorite to severe burn patients increased their rate of survival post infection with Gram-negative bacteria, of which antisepsis cannot be solely responsible.

**Methodology:**

Broth microdilution experiments were performed to confirm anti-microbial activity. Cytokine exposure was performed by dialytic exposure of serum spiked with either interleukin-6 or interleukin-10, accounting for contextually appropriate organic load. Cytokine degradation was measured by immunoassay and by functional bioassay utilising HEK-Blue cells. Hypochlorite mediated cytotoxicity against dermal cells was assessed via direct exposure, followed by a 1-hour attenuation period. Resazurin was utilised to assay the impact of hypochlorite on cell viability.

**Results:**

We have shown that this formulation of hypochlorite in isotonic saline has multiple mechanisms. The first is that 7 mmol/L hypochlorite is capable of eliminating both Gram-negative and Gram-positive bacteria, and that the same concentration differentially degrades the function of IL-6 and IL-10. IL-6 was seen to be 3 times more susceptible to loss of function than IL-10 after only 5 minutes of exposure (*P* < 0.005). The asymmetrical effect on these cytokines was observed between 7 and 1.75 mmol/L, and across the entire time scale examined. This hypochlorite formulation has the capability to sterilize a wound, and by altering the cytokine profile reduce inflammation and scarring, and improve wound healing.

**Conclusions:**

These findings, taken with the burn survival study, suggest that the increased survival rate after application of hypochlorite could be due to the combined effect of elimination of infective pathogens, and the differential degradation of interleukin-6 and interleukin-10 at the site of injury.

## Introduction

The rate at which a wound heals is a critical factor in survival, as the prolonged loss of skin integrity can be associated with considerable morbidity. Two factors which control the rate of closure are the profile of the inflammatory response and incidence of infection. Wound healing is represented as several overlapping phases, with this study focusing on that of inflammation and proliferation. The inflammatory phase is instigated by sentinel cells that secrete cytokines, which are responsible for the attraction and infiltration of the wound area with neutrophils and macrophages [1]. The cytokines present in the inflammatory phase produce a distinct profile whose characteristics can be changed by the presence of infection.

The present study focuses on two cytokines present during inflammation, IL-6 and IL-10. In a wound, IL-6 plays a role in stimulating and maintaining the innate immune response, both locally and systemically, by such actions as recruiting leucocytes to directly eliminate challenges present in the wound [2]. After the acute phase response, IL-6 is one of the cytokines responsible for maintaining the wound in an inflamed state, with overabundance of IL-6 in a wound leading to collateral damage or chronic inflammation [3]. The primary role of IL-10 is reducing the intensity of inflammation to limit this collateral damage to host tissues, and from runaway cascades of both the humoral and innate immune system [4]. To reduce inflammation, IL-10 directly activates anti-inflammatory signals and inhibits the effects of pro-inflammatory cytokines such as IL-6, by blocking their pro-inflammatory signal pathways [5]. However, disorders with IL-10 signaling can cause fatal consequences in addition to chronic disorders. Non-lethal infections of *Toxoplasma gondii* became fatal when IL-10 was knocked out, with these mice showing changes in cytokine release similar to that of septic shock [6].

It is clear that both cytokines are needed in the process of non-sterile wound healing, however, in a sterilized wound a reduction in IL-6 would amplify the effects of any IL-10 present, decreasing the time taken to transition to the next phase of wound healing.

The resolution of an injury is adversely affected by infection, with heavily contaminated wounds taking much longer to heal, becoming chronic wounds, or directly contributing to mortality. Prior to the advent of antibiotic therapy, hypochlorite solutions were often used as topical agents to clean wounds [7]. However, the use of hypochlorite solutions on wounds declined as the mainstream use of antibiotics climbed. The increased use of these drugs has caused the rapid emergence of antimicrobial resistance (AMR), which has since become a major concern in care centers where antibiotic use is critical to patient care, such as in burns and intensive care units [8]. There is a clear case for hypochlorite being an antibiotic sparing therapeutic, one which no bacteria have been known to form resistance against, forming a key tool to tackle the AMR crisis [9].

In 2024, one burn unit applied a topical sodium hypochlorite solution as part of the treatment for patients who experienced Gram-negative infections. Patients in the treatment group showed greater 90-day survival rates compared with those of the same burn severity who received the normal standard of care [10]. These improved survival rates are unlikely to be rooted solely to the removal of bacterial contamination; modulation of the inflammatory response is most likely to occur, with direct effects upon wound healing.

Previous work has demonstrated that the concentration and complexity of organic load can significantly reduce the efficacy of hypochlorite in eliminating bacteria [11,12]. For example, Pappen et al. indicated that increasing the concentration of BSA increases the concentration of hypochlorite necessary to kill bacteria effectively [13]. Suggesting that in a clinical setting the presence of organic load would reduce the effectiveness of hypochlorite [13]. As such, the presence, concentration, and components of the organic load faced in vivo needs to be considered.

The current favoured method of quenching the oxidative qualities of hypochlorite is to provide a reducing agent, such as sodium thiosulfate [14]. However, this has the potential to interfere with downstream testing that involves reducing or oxidizing agents. As such, dialysis was considered as the method for controlled exposure of cytokines to hypochlorite, whilst in the presence of an organic load that accurately mimics the environment present in a dermal wound. In such a wound cytokines would not come into immediate direct contact with hypochlorite, rather the serum hosting the cytokines would have a significant effect on hypochlorite action, hence the spiking of cytokines in heat-inactivated serum.

A hypochlorite volume excess of 1000-fold would mimic a constant irrigation of the wound by the hypochlorite solution. Combining to create a system that represents a wound being treated with hypochlorite.

With this aim in mind, dialysis is also a more appropriate method than chemical quenching, as that would not be performed in a clinical setting.

The present study demonstrates the differential degradation of IL-6 and IL-10 following their exposure to hypochlorite in the presence of an organic load. IL-6 loses function and structure faster than IL-10. Further investigation provided insights into the tolerability of skin to this formulation of hypochlorite in saline and highlighted the importance of managing organic load. These results reveal the mechanism of action behind the anti-inflammatory nature of hypochlorite and suggest a role for specific concentrations of this chemical in wound care, by sterilising the wound of infective agents, reducing the severity of the immune response, aiding in wound closure, and reducing scarring.

## Materials and methods

### Establishment of minimum inhibitory and bactericidal concentrations

The following Gram-positive strains were used: *Bacillus subtilis* (8054), *Enterococcus faecalis* (ATCC 51299), *Staphylococcus aureus* (43300 and 6571). With the following Gram-negative strains being used: *Escherichia coli* (12923), *Klebsiella pneumoniae* (13883) and *Pseudomonas aeruginosa* (ATCC 27853). All were cultured in Mueller Hinton broth (MHB, Sigma-Aldrich, Missouri, USA) or on Nutrient agar (NA, Formedium, Norfolk, UK). The concentration of bacteria used were measured by comparison to a 0.5 McFarland standard (Remel, Kansas, USA) ending with a bacterial concentration of 1 × 10^6^ CFU/mL in MHB. Seeding densities were confirmed by a 1:1000 dilution of bacterial samples inoculated onto NA and incubated at 37 °C overnight. The colonies were counted and checked against expected seeding density. A broth microdilution method was employed to ascertain the minimum inhibitory concentration (MIC) of this hypochlorite formulation. The hypochlorite solution was serially diluted in sterilized saline (0.9%) from 7 mmol/L to 13.7 µmol/L, equal volumes of inoculum and hypochlorite or saline were combined and incubated at 37°C for 24 hours. These were checked visually for evidence of growth, with technical and biological triplicates being performed for each strain. Each well showing no growth and the highest concentration showing growth were inoculated onto NA and incubated for a further 24 hours to determine the minimum bactericidal concentration (MBC).

### Dialysis and measurement of inflammatory cytokines

IL-6 and IL-10 (Peprotech, New Jersey, USA) were reconstituted following manufacturer's instructions, and then further diluted to 50 ng/mL in FBS (Life Science Production, Bedfordshire, UK) that had been heat inactivated at 60°C for 45 minutes (HIFBS). Dialysis was performed to expose the cytokine-spiked HIFBS to hypochlorite for 5–30 minutes by filling a 3.5 kDa MWCO dialysis cassette with 5 mL of inoculated HIFBS, and adding this to 5L of dialysis fluid. After dialysis, remaining hypochlorite was quenched by dialysis of the cassette against 5L of 0.9% (w/v) saline for a minimum of 30 minutes. Control samples were not dialysed against hypochlorite, and instead dialysed against 0.9% (w/v) saline for 30 minutes. The resulting samples measured in triplicate either by ELISA (RnD Biosystems, Minnesota, USA) according to the manufacturers protocol or using a functional assay using the HEK-Blue cell line (HKB-IL6 and HKB-IL10 (Invivogen, California, USA)) according to the manufacturer’s instructions.

### Cytotoxicity

Cytotoxicity of the hypochlorite in saline solution was determined using human foreskin fibroblasts (HFF-1, ATCC SRC-1041). Measurement was made either in the presence of 10% FBS to equate to organic load or in its absence. A positive kill control of 1 × working strength disinfectant (Chemgene, Merseyside, UK) was used. Cells were exposed to different concentrations of hypochlorite in saline for 5 minutes, washed, then incubated for one hour and then 5 µL of resazurin (1 mg/mL, Sigma-Aldrich, Missouri, USA). The plate was incubated for 2 hours and measured for absorbance at 570 nm and 600 nm and viability determined following the manufacturer’s protocols (Bio-Rad, California, USA). The mean percentage viability for the 4 biological replicates were plotted and a Boltzmann sigmoidal curve was fitted to the data, and the IC_50_ value calculated.

### Statistical analysis

Both the ELISA and functional assay results were processed as follows: after performing the necessary computations as described by the manufacturer, percentage interleukin activity was calculated by subtracting the control sample values from the experimental values.

The results of the descriptive analysis are presented for numerical variables in the form of averages, sample sizes and percentages were calculated for categorical outcomes. Student’s *t*-tests were performed with Bonferroni corrections to compare the groups listed in the results section.

## Results

### Establishing the minimum inhibitory and bactericidal concentrations

The MIC and MBC for sodium hypochlorite in saline were established for a range of Gram-negative and Gram-positive bacteria associated with common wounds and burns (Table 1). All species of bacteria tested showed the same susceptibility to this formulation of hypochlorite with all bacteria showing total bacterial death after exposure to 7 mmol/L sodium hypochlorite in saline, including methicillin resistant *S. aureus* (MRSA).

**Table 1:** Description of the MIC and MBC of several bacterial species when exposed to hypochlorite and saline.

Gram-status	Species/Strain	MIC (mmol/L)	MBC (mmol/L)
Positive	*E. faecalis*	7	7
	*B. subtilis*	7	7
	*S. aureus 6571*	7	7
	*S. aureus 43300 (MRSA)*	7	7
Negative	*E. coli*	7	7
	*K. pneumoniae*	7	7
	*P. aeruginosa*	7	7

Minimum inhibitory and bactericidal concentrations of bacteria commonly found in contaminated wounds for sodium hypochlorite 7 mmol/L in saline.

Identification of 7 mmol/L hypochlorite in saline needed for the MIC and MBC prompted the use of this concentration as a starting point for analysis of the effects on the cytokines IL-6 and IL-10.

### Effects of exposure on IL-6 and IL-10

**Fig 1:**
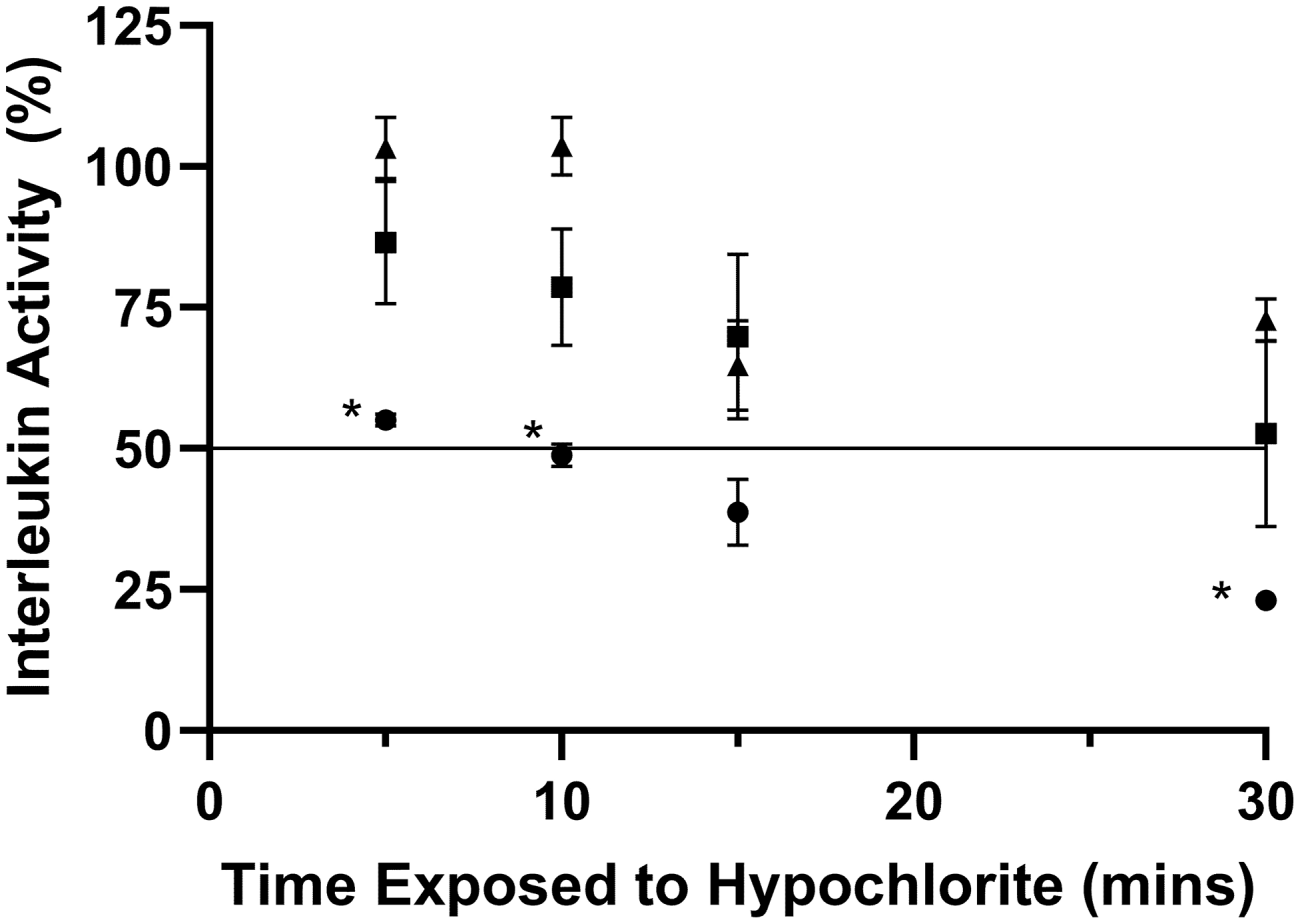
IL-6 activity reduction by hypochlorite in saline at three concentrations, over 30 minutes. Activity was assessed by ELISA, with all data normalised against cytokine not exposed to hypochlorite. Mean and standard deviations are displayed, and two-tailed student’s t-tests were used to establish significant differences between 0 minutes and each time point, for each concentration. (* P ≤ 0.05, N = 3) ● 7 mmol/L hypochlorite ■ 3.5 mmol/L hypochlorite ▲1.75 mmol/L hypochlorite.

Exposure of either cytokine to hypochlorite in saline led to loss of structure and function in a time and dose dependent manner. Structural changes identified by ELISA (Fig 1) revealed that hypochlorite in saline at 7 mmol/L led to a significant reduction in IL-6 (*P* = 0.04) after 5 minutes.

Exposure of IL-10 to hypochlorite in saline at different concentrations showed similar results to IL-6 across time and concentrations, with a decrease in the intensity compared to IL-6. A rapid, significant change in cytokine structure was seen at 7 mmol/L (*P* = 0.04). Whereas 3.5 and 1.75 mmol/L hypochlorite caused no significant degradation of IL-10 structure (*P* > 0.05).

The intensity of this degradation reduced as the concentration lowered. At a concentration of 1.75 mmol/L, the reduction in IL-6 was at its lowest with none of the timed samples showing significant decline from the original (*P* > 0.05).

**Fig 2:**
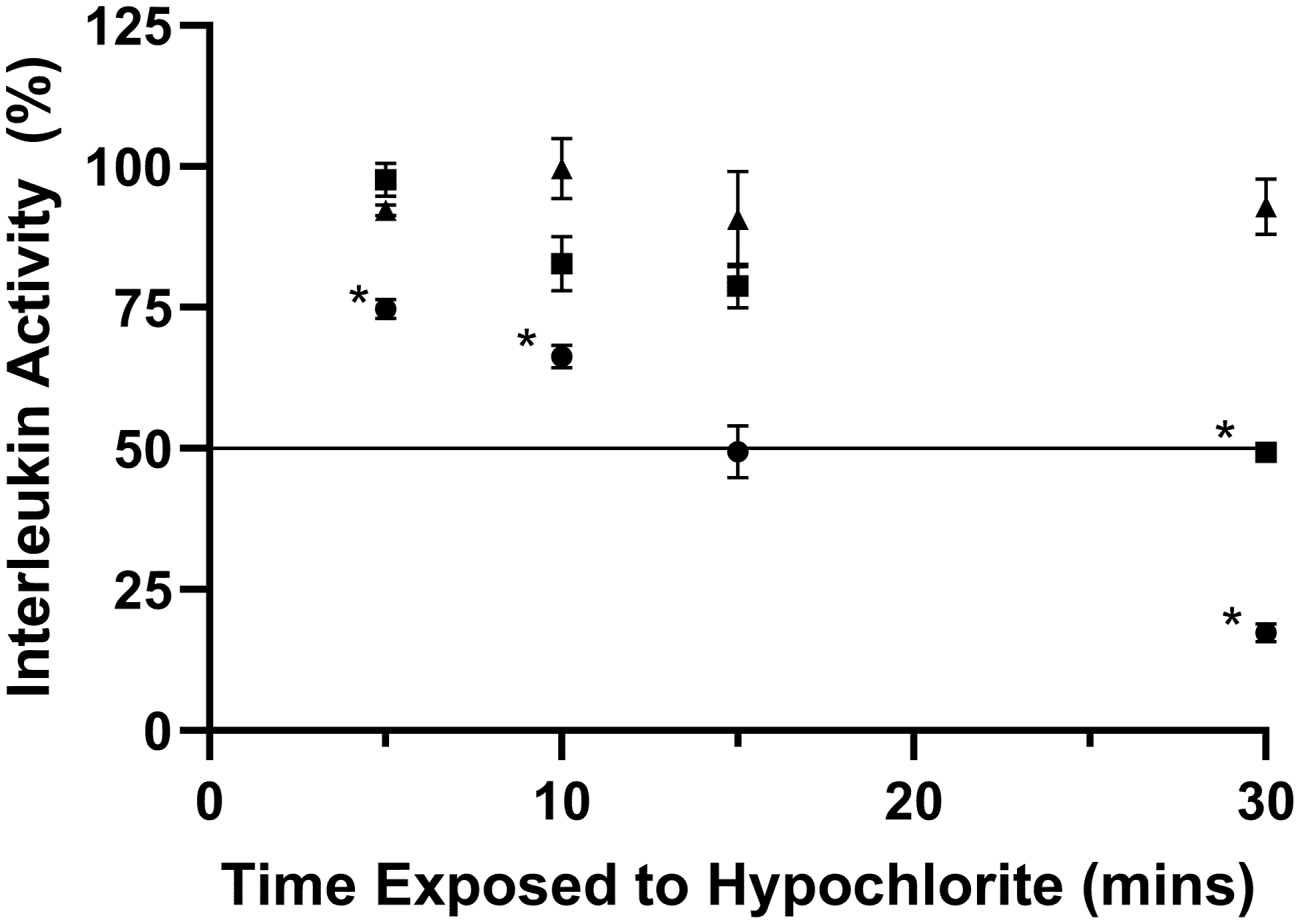
IL-10 activity reduction by hypochlorite in saline at three concentrations, over 30 minutes. Activity was assessed by ELISA, with all data normalised against cytokine not exposed to hypochlorite. Mean and standard deviations are displayed, and two-tailed student’s t-tests were used to establish significant differences between 0 minutes and each time point, for each concentration. (* P ≤ 0.05, N = 3) ● 7 mmol/L hypochlorite ■ 3.5 mmol/L hypochlorite ▲1.75 mmol/L hypochlorite..

Fig 2 demonstrated that the exposure of IL-10 to hypochlorite in saline at different concentrations showed similar results to IL-6 across time and concentrations, with a decrease in the intensity compared to IL-6. As with the previous assay, there are initial strong changes seen at 7 mmol/L (*P* < 0.05), with this effect diminishing at lower concentrations (*P* > 0.05).

### Bioassays

**Fig 3:**
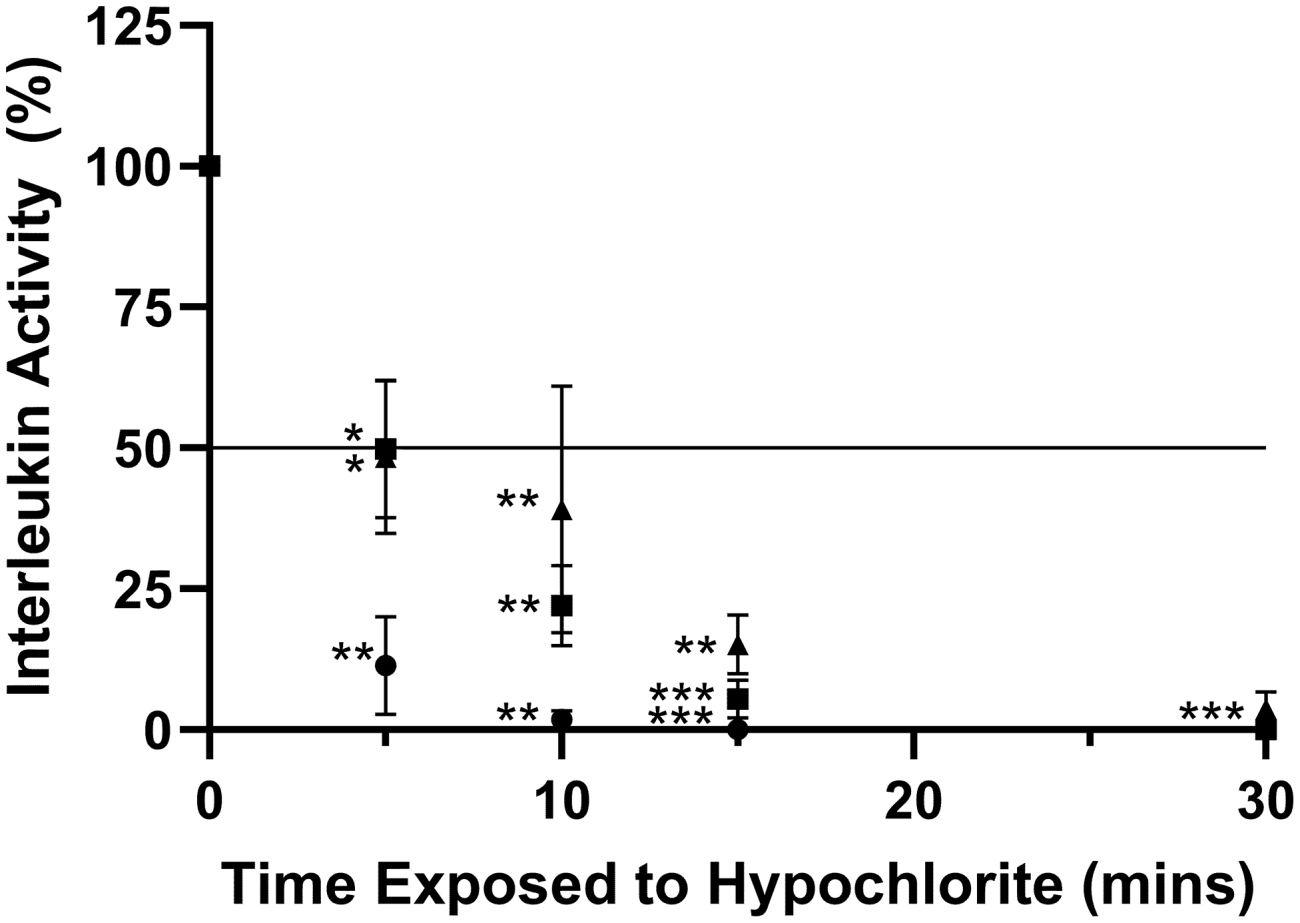
IL-6 activity reduction by hypochlorite in saline at three concentrations, over 30 minutes. Activity was measured by functional HEK-Blue cell lines, with all data normalised against cytokine not exposed to hypochlorite. Mean and standard deviations are displayed, and two-tailed student’s t-tests were used to establish significant differences between 0 minutes and each time point, for each concentration. (* P ≤ 0.05, ** P ≤ 0.01, *** P ≤ 0.001, at 30 minutes all P ≤ 0.001, N = 3) ● 7 mmol/L hypochlorite ■ 3.5 mmol/L hypochlorite ▲1.75 mmol/L hypochlorite.

**Fig 4:**
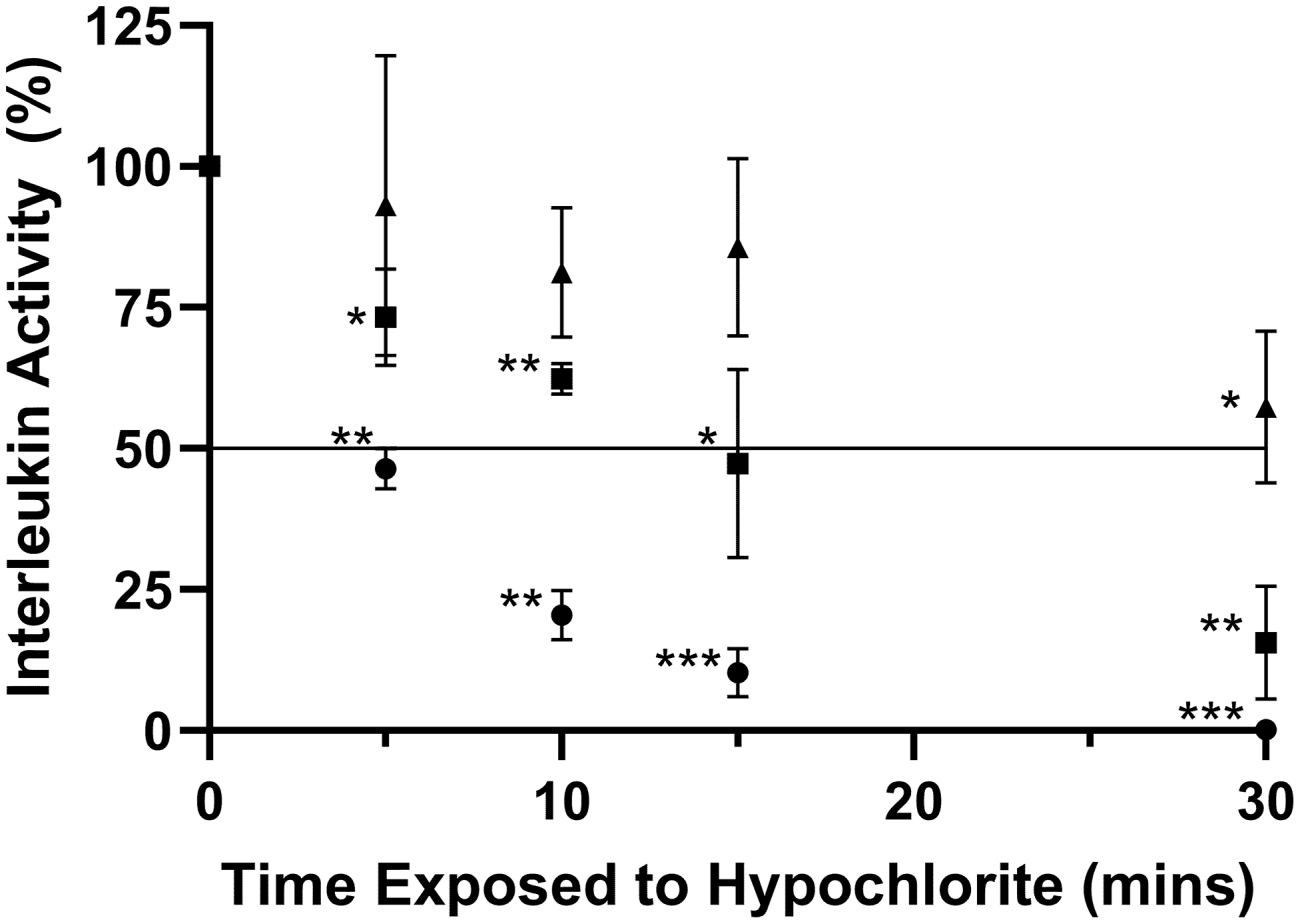
IL-10 activity reduction by hypochlorite in saline at three concentrations, over 30 minutes. Activity was measured by functional HEK-Blue cell lines, with all data normalised against cytokine not exposed to hypochlorite. Mean and standard deviations are displayed, and two-tailed student’s t-tests were used to establish significant differences between 0 minutes and each time point, for each concentration. (* P ≤ 0.05, ** P ≤ 0.01, *** P ≤ 0.001, N = 3) ● 7 mmol/L hypochlorite ■ 3.5 mmol/L hypochlorite ▲1.75 mmol/L hypochlorite.

Whilst the pattern of degradation mirrors that seen in the ELISA dataset, the functional bioassays of exposed IL-6 and IL-10 revealed greater reductions in function than those in structure (Fig 3 and 4).

**Fig 5:**
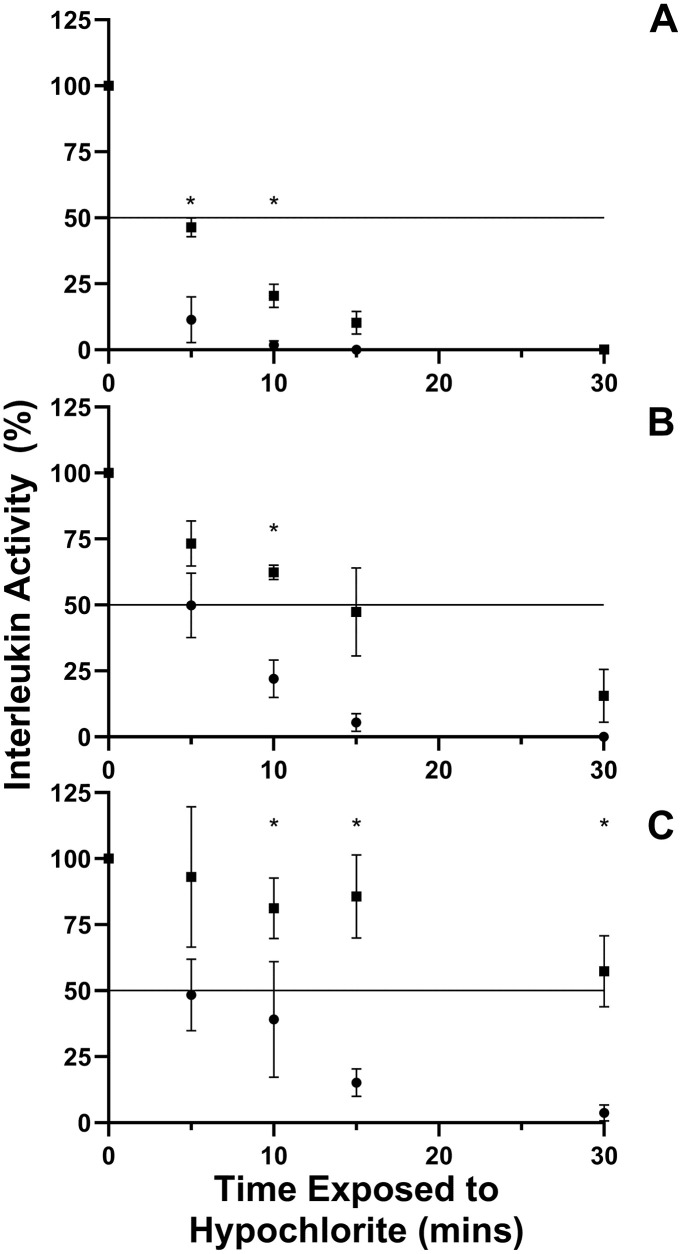
Comparing the degradation of activity between IL-6 and IL-10 measured by functional HEK-Blue cell lines. All data normalised against cytokines not exposed to hypochlorite. Mean and standard deviations are displayed, and two-tailed student’s t-tests were used to establish significant differences at each time point between each cytokine (* P ≤ 0.05, N = 3) ● IL-6 ■ IL-10.Hypochlorite at: A – 7 mmol/L, B- 3.5 mmol/L, C – 1.75 mmol/L.

The anti-inflammatory nature of this hypochlorite formulation is shown in the comparison of the effects of hypochlorite exposure on IL-6 and IL-10 (Fig 5). It was revealed that at all concentrations tested there was significantly greater reductions in the functional activity of IL-6 compared with IL-10, with the same true at most time points. The effect was most pronounced at 7 mmol/L, the same concentration at which all bacteria tested were eliminated.

### Cytotoxicity of hypochlorite to HFF-1 cells in the presence of organic load

**Fig 6:**
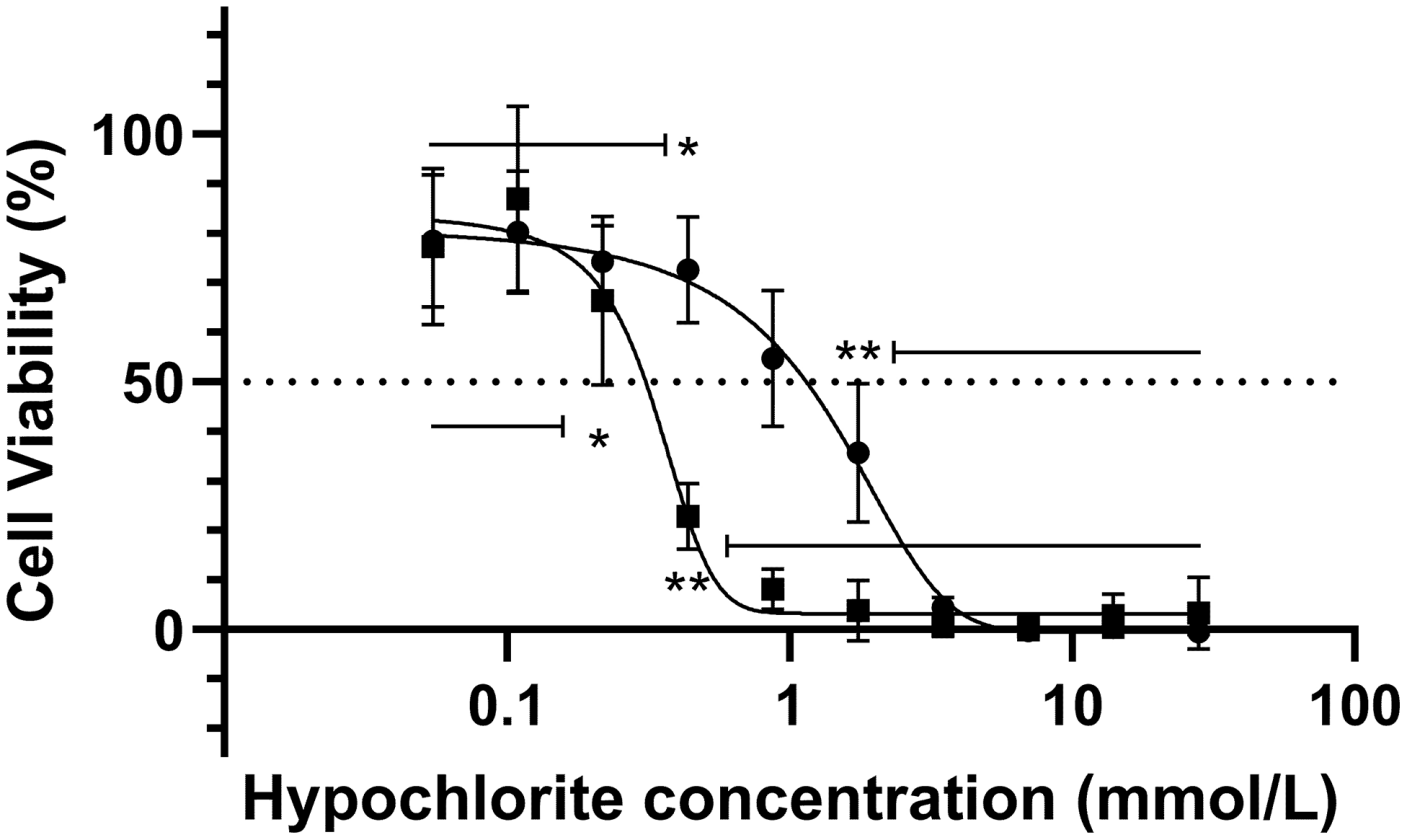
HFF-1 viability when exposed to isotonic hypochlorite in the presence and absence of organic load. * = The first statistically significant reduction from 80% viability. ** = The last statistically significant viability difference from 0% viability. All when utilising paired, two-tailed student’s t-tests (P ≤ 0.05) ● Organic load ■ No organic load.

The highest concentration of hypochlorite in saline that HFF-1 cells could tolerate with no significant reduction in cell viability was 0.22 mmol/L (*P* = 0.06). The viability then trended downwards, passing the IC50 value of 1.15 mmol/L. The highest concentration at which the HFF-1 cells could survive was 1.75 mmol/L (*P* = 0.047). For concentrations higher than this, there was no significant difference in viability from 0 (Fig 6).

Without organic load present, the cells showed a much higher sensitivity to hypochlorite. The first loss of viability occurred at 0.22 mmol/L (*P =* 0.026). As with the organic load experiment, viability then decreased as the concentration increased. The IC_50_ in the absence of organic load was 0.311 mmol/L. lastly, the highest concentration of hypochlorite that HFF-1 cells could survive without organic load was 0.44 mmol/L (*P =* 0.048).

## Discussion

This is the first identification of a formulation of hypochlorite in saline that is capable of both eliminating multiple key species of bacteria, as well as modulating key cytokines in the hyper-inflammatory response. Taking a first step in investigating the mechanism behind the survival benefit that is conferred to burn patients when treated with a similar solution. Other mechanisms behind improved outcomes for the burn patients must still be addressed. This includes investigating chemotaxis as a result of hypochlorite treatment. However, it is likely that the removal of bacteria, and the modulation of inflammation, are both major contributors to the improved survival rates.

Adverse components of inflammation include bacterial contamination and a high IL-6:IL-10 ratio. Here we show that hypochlorite at defined concentrations can act as a bacteriolytic agent against both Gram-negative and Gram-positive organisms whilst functionally inactivating IL-6 at a rate greater than IL-10. Consequently reducing the probability of scarring, cytokine release syndrome, and formation of chronic wounds [3,15]. The differential response of HFF-1 cells to hypochlorite in the presence and absence of HIFBS indicates the necessity and successful management of organic load.

Hypochlorite is an effective anti-bacterial agent, killing a broad spectrum of bacteria through the aggregation of essential proteins [16]. We have shown that 7 mmol/L hypochlorite is the minimum concentration needed to eliminate both Gram-negative and Gram-positive bacteria including *S. aureus*, one of the most prevalent infections in intensive care units and associated with a high risk of bacterial sepsis [17].

In a wound, IL-6 is a pro-inflammatory cytokine, whereas IL-10 is an anti-inflammatory and pro-resolution cytokine [3,4,18]. Our results indicate both structural and functional changes in IL-6 and IL-10. The former is in line with previous work where hypochlorite was shown to degrade IL-6, specifically at methionine 161 and tryptophan 157, both of which have been implicated in receptor binding of the cytokine [14]. Our data reveals that the rate and intensity in loss of both structure and function was not the same in both cytokines with IL-10 seemingly more resilient to hypochlorite degradation than IL-6.

In vivo reductions in IL-6 function and the comparative retention of IL-10 function would lead to a reduction in pro-inflammatory signaling, with anti-inflammatory signals remaining. The result of this is expected to be reduced acute inflammation which in turn would lower the chance of cytokine release syndrome, along with causing an accelerated transition into a wound healing state. As seen previously in human volunteers, model wounds treated with 2.3 mmol/L hypochlorite progressed through the phases of wound healing faster than control wounds [19]. In addition, they showed that by day 4, the bacterial burden of the wounds was reduced after treatment with hypochlorite, and between treatments. This further confirms the suitability of hypochlorite as a topical antiseptic that promotes wound healing and provides immediate relief of bacterial burden.

A third mechanism of action of hypochlorites suggested by this work is reducing the incidence and severity of scarring. Previous studies revealed that patients treated with hypochlorite post-surgery experienced reductions in pathological and non-pathological scarring [20]. Our results would suggest that this could be due to the asymmetric effect of hypochlorite on IL-6 and IL-10. An overabundance of IL-6 has been implicated in significant increases in scar formation, and overexpression of IL-10 has been shown to restore the fetal phenotype of scarless wound healing in adult mouse skin [3,20,21].

One million activated neutrophils can generate 20 µmol/L of hypochlorous acid during a 2-hour incubation [22] suggesting that human tissue is capable of tolerating concentrations in this amount. This is extended by the present study, where it was found that a monolayer of skin fibroblasts can tolerate up to 220 µmol/L without significant reductions in metabolism.

In a clinical setting, the patient’s body forms a near infinite source of organic load, and this must be considered in experimentation [11,13]. As in vivo the same effect would be seen in a wound, the surrounding healthy tissue would act as organic load, reducing the toxicity of the hypochlorite to the living tissue within the wound. This would leave dead tissue to be dissolved, bacteria to be lysed, and extracellular components such as IL-6 and −10 to be degraded [23].

## Conclusion

The present paper demonstrates the differential response of IL-6 and IL-10 to hypochlorite, in the concentration range of 7 and 1.75 mmol/L and in a manner which accommodates a context specific organic load. Showing that not all inflammatory mediators are created equal in the eyes of hypochlorite. The consequences of this difference in a wound treated with hypochlorite could be a marked reduction in inflammation due to the removal of a key pro-inflammatory agent, and the conservation of an anti-inflammatory agent. Further pro-resolution effects of hypochlorite use could be the elimination of bacteria and necrotic tissue, further reducing inflammation.

Secondly, we have demonstrated the first use of dialysis to expose hypochlorite to inflammatory factors. This method allows the exposure of proteins to known concentrations of hypochlorite, in the presence of an optimizable organic load, for fixed periods of time. This method could be further expanded to include exposure subjects including suspension cells in media, blood, bacteria, or other clinically relevant proteins.

The use of hypochlorite solutions of fixed concentrations, in the presence of an un-buffered isotonic saline could benefit the clinic vastly. This paper, in the context of other publications in the field of hypochlorite research, has indicated that this formulation is a suitable antiseptic, against a range of bacteria, with no indications of resistance forming. This action leads to hypochlorite being a suitable replacement for antibiotics for topical infections or abiotic prophylactics.
